# The AGE Effect on Protective Behaviors During the COVID-19 Outbreak: Sociodemographic, Perceptions and Psychological Accounts

**DOI:** 10.3389/fpsyg.2020.561785

**Published:** 2020-10-16

**Authors:** Rita Pasion, Tiago O. Paiva, Carina Fernandes, Fernando Barbosa

**Affiliations:** Laboratory of Neuropsychophysiology, Faculty of Psychology and Educational Sciences, University of Porto, Porto, Portugal

**Keywords:** pandemic (COVID-19), aging, risk, protective behaviors, anxiety, fear, social isolation, optimism

## Abstract

COVID-19 outbreak is a sudden and devastating global pandemic in which the control of the spread is highly dependent on individual reactions, until the development of a vaccine and adequate treatments. Considering that older adults are at high risk for COVID-related medical complications and mortality, the present study focuses on the age-related differences on the adoption of protective behaviors during the initial stages of this outbreak, while accounting for the role of sociodemographic, COVID-related, perceived risk, and psychosocial variables (i.e., anxiety, optimism, fear of death, and social isolation) in this relation. The study sample included 1696 participants, aged between 18 and 85 years old, who completed an online survey during the initial stages of the first COVID-19 outbreak in Portugal. Overall, results reveal that the engagement in protective behaviors declines with advancing age and that older adults show a pattern toward lower perceived risk compared with middle-aged adults. Multicategorical mediation analyses show that anxiety, optimism, fear of death, and social isolation significantly mediate age effects on protective behaviors. Specifically, both anxiety and fear of death increase protective behaviors via higher perceived risk in the middle-aged and in the younger groups, respectively. Optimism directly predicts protective behaviors in the middle-aged groups, while social isolation reduces protective behaviors in the younger and older-aged groups. Results are discussed in terms of its implications for public health policies.

## Introduction

On December 31, 2019, the World Health Organization (WHO) was informed of a cluster of cases of severe acute respiratory syndrome of unknown etiology in Wuhan, Hubei province of China (WHO, 2020a). A novel coronavirus–the SARS-CoV-2–was identified as the cause of the COVID-19 respiratory disease. On March 11, COVID-19 was officially recognized as a global pandemic and was followed by calls for governments’ actions to stop the spread of the virus (WHO, 2020b).

In European Union, the first cases were reported on January 25 (WHO, 2020c). 2 months later, there were more than 1.000.000 of confirmed cases and 100.000 deaths with Spain, Italy, France, and Germany being the most affected countries. Despite the official mitigating measures (e.g., closure of borders, non-essential services, and schools, appeal for teleworking and voluntary home curfew, declaration of states of emergency), the spread of a virus seems to be highly dependent on rapid changes of population’ behavior, namely in what regards the engagement in protective behaviors, such as hygiene practices and social distancing (Bish and Michie, 2010; Wise et al., 2020). Thus, the individuals’ ability to perceive the risks associated with virus transmission is of critical importance to boost protective behaviors during the outbreak.

A review of 26 studies (Bish and Michie, 2010) demonstrated that the perceived vulnerability of becoming infected shapes indeed protective behaviors. Those reporting higher perceived risk during SARS 2003 and H5N12004 outbreaks seem to be more likely to take precautionary measures against the infection (Leung et al., 2003; Brug et al., 2004; Fielding et al., 2005; Tang and Wong, 2005). Another recent review of 14 studies (Webster et al., 2020) showed that individuals who perceived SARS, Ebola, and H5N1 to be riskier in terms of transmission and severity adhered to a greater extent to the quarantine, especially at the second outbreak wave. Seminal research on COVID-19 further revealed that protective behaviors (e.g., to wash hands and to stay at home) were more frequent 5 days after the first assessment due to growing risk awareness (Wise et al., 2020).

From the evidence on a link between perceived risk and protective behavior, a necessary second step is to search for risk/protective factors that may be mediating and moderating this relation. The current study focuses on aging and psychological individual differences effects in risk perceptions and protective behaviors during the first days of the COVID-19 outbreak. The relevance of these factors for a comprehensive understanding of risk-taking during epidemics, especially in those at high-risk for medical complications and mortality, will be discussed below.

### Aging

In Europe, 18% of the population has more than 65 years old (Population Reference and Bureau, 2020). At 65 years, European citizens could expect to live about an additional 20 years and the number of centenarians is projected to be more than half a million by 2050 (Eurostat, 2019). The structural process of demographic aging poses several challenges during outbreaks in which the older groups are at high-risk for medical complications and mortality. Although all age groups can contract COVID-19, individuals aged above 65 years face more risks of developing severe illness, especially due to cumulative health conditions that are likely to come with aging (European Centre for Disease Prevention and Control, 2020). Subsequently, it is of critical importance to assess to what extent the elderlies feel more susceptible to being affected by COVID-19, and how this perception, alongside some psychological processes, affects their commitment with quarantine and protective behaviors.

Although it is commonly assumed that older adults are more risk-averse than their younger counterparts, the results are mixed. Older adults report lower levels of impulsivity and sensation-seeking (e.g., Spinella, 2007), but do not differ in pathological gambling rates (Welte et al., 2001). One explanation for this inconsistency is that attitudes toward risk are not a single trait but rather an interaction between individual differences and specific situations (Bonem et al., 2015).

Previous studies conducted during epidemics do not provide clear evidence on this matter. Some studies demonstrate that elders were more likely to undertake appropriate measures against SARS 2003 (e.g., Lau et al., 2003; Leung et al., 2003; Vijaya et al., 2005), while others indicate that older individuals are less likely to follow the recommendations for preventing SARS (Wong and Tang, 2005) and H1N1 (Rubin et al., 2009). For instance, elders perceived lower risk from buying live chickens in the H5N1 epidemic (Fielding et al., 2005), and seemed to not intend to be vaccinated (Bish and Michie, 2010). Finally, there are studies reporting no relation between age and protective behaviors in both SARS 2003 affected (Tang and Wong, 2005) and non-affected areas (Brug et al., 2004).

From this standpoint, the current work intends to analyze the role of risk perceptions on protective behavior as a function of aging during the COVID-19 outbreak. The first evidence on COVID-19 revealed that age does not moderate the link between risk perceptions and protective behaviors (Wise et al., 2020), but these conclusions were retrieved from a younger sample. Considering that relations can be complex, and the results may not be straightforward, the current study further explores group differences in relevant sociodemographic characteristics (e.g., education, health problems, traumatic experiences) and COVID-related variables (e.g., access to information, similar symptoms in the past days, diagnosis among one’s acquaintances). Additionally, psychological dimensions are good candidates to deepen our knowledge on aspects mediating preventive measures.

Aging is associated with a “positivity effect” on cognitive and affective processing. That is, older adults exhibit a decline in the processing of negative stimuli compared to the younger counterparts, with intact or enhanced processing of positive stimuli (see Mather, 2016). Such findings have been interpreted within the framework of socio-emotional selectivity theory, whereby changing time horizons may lead to the prioritization of emotionally relevant goals (Charles and Carstensen, 2010). Considering that cognitive and affective processing may modulate risk perceptions and protective behavior, our goal is to unveil the relations between aging and individual differences on psychological dimensions related to positive and negative affect during the initial stages of the COVID-19 outbreak (i.e., first period of the mandatory quarantine for all the national citizens during the Emergency State).

### Anxiety

Previous studies on SARS, H1N1, and H5N1 reveal that moderate levels of anxiety can lead to appropriate preventive responses to avoid risky behaviors (Leung et al., 2003; Vijaya et al., 2005; Rubin et al., 2009; Bish and Michie, 2010), probably due to higher perceived risk in anxious individuals (Fielding et al., 2005; Vijaya et al., 2005). In the COVID-19 outbreak, Wang et al. (2020) showed that about one-third of the participants reported moderate-to-severe anxiety and that the preventive behaviors of the last two weeks reduced anxiety levels. That is, the effects seem to be recursive: higher levels of anxiety may foster the practice of caution behaviors in the first stage, which will reduce the worries about contamination later. Nonetheless, the balance for an “adaptive anxiety” to this context is delicate: excessive anxiety triggers panic reactions that are often disproportional to the real risks, while the lack of anxiety brings inertia for prevention (Leung et al., 2003). In this line, the decline of negative affect in the elders, such as anxiety levels, may be a risk factor for decreasing risk perceptions and, consequently, protective behaviors.

Epidemiologic surveys have systematically found that current and lifetime anxiety disorders are less prevalent in older than younger adults (e.g., Kessler et al., 2005), a finding that is independent of race, marital status, cognitive function, and medical comorbidity (Flint et al., 2010). However, one study on COVID-19 found that individuals under 18 years old had the lowest scores on psychological distress (i.e., anxiety and depression), while younger and older groups aged above 60 years reported the highest scores (Qiu et al., 2020). The authors proposed that the older group may be more concerned about their survival. Wang et al. (2020) pointed out indeed that the history of chronic illness, but not age, emerged as the main predictor of anxiety during this epidemic. From these results, it is important not only to gather evidence on how anxiety modulates risk assessment and preventive conducts but also to clarify how individual characteristics expected to co-vary with age may act either as risk or protective factors. For example, age-related health problems may increase cautionary attitudes, but lower educational levels and previous traumatic experiences in this population are expected to reduce protective behavior (Fielding et al., 2005; Bish and Michie, 2010).

### Fear of Death

The fear of death is a natural phenomenon during outbreaks. The number of deaths increases exponentially every day, and the acute and severe nature of the disease, as well as the uncertainty around the illness outcomes, inherently raises concerns around death (Sze and Ting, 2004; Mok et al., 2005). For instance, people who survived Ebola 2013–2016 and SARS 2003 epidemics tend to disclosure more fear of death (Mok et al., 2005; Van Bortel et al., 2016). Moreover, Wang et al. (2020) reported that a lower perceived chance of surviving to COVID-19 if infected was associated with higher levels of stress. Thus, the fear of death may be increased in groups at higher risk for mortality and may emerge as a protective factor for engaging in preventive measures (Sze and Ting, 2004).

Yet, paradoxically, the oldest of the elderly report no fear of death (Johnson and Barer, 1997). The large body of literature suggests that this fear is greater among younger adults, peaking around middle age and declining with aging (Fortner et al., 2000; Thorson and Powell, 2000; Cicirelli, 2002; Russac et al., 2007). Despite needing further investigation, the reduction of a negative affective state as fear of death may reduce the influence of health concerns and modulate older adults’ attitudes toward the COVID-19 pandemic.

### Optimism

To anticipate the future is a critical aspect to guide behavior, namely in new situations as outbreaks. One of the most consistent findings is that our brain is not accurate when making inferences about the future. Humans tend to overestimate the probability of positive events and underestimate the negative ones, which is particularly true for health problems (Weinstein, 1984; Chapin, 2001; Sharot, 2011). Individuals tend to think that their chances of having health problems are lower than their peers (Weinstein, 1984). Even in the face of negative disconfirming evidence, there is a resistance to change the optimistic expectation (Sharot, 2011). From an evolutionary perspective, this bias is adaptive to human life (e.g., expecting positive outcomes reduces anxiety and increases performance). Greater optimism is associated with exceptional longevity (Lee et al., 2019), and with the maintenance of healthy aging over time (Kim et al., 2019). However, the underestimation of risks may reduce protective behaviors essential for survival and, subsequently, the vulnerability to such hazards (Weinstein, 1984; Sharot, 2011). This means that excessive optimism may generate reactions based on a perception that does not match the real outbreak scenario.

In a SARS 2003 unaffected area, Brug et al. (2004) evidenced that only 5% of the individuals were worried about becoming infected by SARS themselves in the future. Although SARS is an infectious disease, the participants estimated the chances of becoming infected as lower than having a heart attack or cancer. In accordance with previous findings, this percentage was slightly higher when assessing the risk for their families (8.3%), with 33% of the respondents rating their risk as being smaller than for their peers of the same sex and age. Wise et al. (2020) also found that participants underestimated their risk for COVID-19 infection compared to the average person in the country. Importantly, 5 days later, the researchers observed rapid increases in the perception of own’s risk, which were driven by more realistic perspectives and lead to meaningful outcomes in terms of reducing risky behaviors for transmission (Wise et al., 2020). This suggests that as the outbreak progresses and the threat gets closer, individuals became more aware of the possibility of getting infected and of the severity of the outcomes, probably because awareness raises from records of diagnosis among acquaintances, from checking similar symptoms, and from the availability of information from media and social networks that ease instances to be recalled and brought to mind (Pachur et al., 2012).

Few studies to date have addressed the effect of age on optimism, and the results are inconsistent (Chowdhury et al., 2014). For instance, one study found that younger, rather than older adults, outlook future with more optimism (Lachman et al., 2008), while an increase in dispositional optimism was observed in a sample aged from 55 to 99 years (Lennings, 2000). Of importance to this topic, Chapin (2001) uncovered a negative association between age and self-protective pessimism toward health risks. This former evidence suggested that variations in a positive affective state, such as optimism, may shape older adults’ propensity to risky behaviors in a pandemic context.

### Social Isolation

Social connection among conspecifics is a defining characteristic of humans as social species and thus the lack of stable social bonds naturally threatens human life (Cacioppo and Cacioppo, 2014). Holt-Lunstad et al. (2015) demonstrated that measures of social isolation and loneliness are associated with increased rates of mortality (about 30%). Despite inconsistent findings (e.g., Cacioppo and Hawkley, 2003), previous studies suggest that these outcomes may be explained, at least partly, via the absence of health-promoting behavior co-occurring with social isolation and feelings of loneliness (Lauder et al., 2006; Hawkley and Cacioppo, 2010; Holt-Lunstad et al., 2015; Hämmig, 2019).

Individuals who are socially disconnected are less exposed to multiple sources of information and normative pressures from their relatives (Cacioppo and Hawkley, 2003), which can minimize the adoption of protective behaviors. Additionally, those individuals lacking support seem to be less motivated to adhere to socially defined standards (Lauder et al., 2006; Hawkley and Cacioppo, 2010). As a result, the actual or perceived social connection may accelerate health-promoting behaviors during pandemics.

In Europe, 2 to 16% of the adult population has no one to ask for help if they need it, and over 1 in 10 persons aged 65 or more has no interaction whatsoever with friends, either personally or in other ways (Eurostat, 2010). Considering that the risk for social isolation increases with age, older adults can easily develop unhealthy habits (Novotney, 2019). Those without social support will further need to interrupt the quarantine to get supplies more often and, consequently, will be more exposed to COVID-19 (Jones, 2020). Thereby, it is important to identify the risks of social isolation among older adults, and how social isolation may influence older adults’ disability to behave safely during pandemics.

### Current Study

The emergence of risk perceptions and protective behaviors during outbreaks might interact with aging and a set of psychosocial dimensions associated with positive and negative affect. From the current state of the art, the present study aims: (1) to analyze risk perceptions and the frequency of protective behaviors in older adults during the initial stages of the COVID-19 in Portugal, (2) to explore age-group differences in sociodemographic characteristics (e.g., educational level, health problems, traumatic experiences) and COVID-19 awareness (e.g., information exposure, similar symptoms in the past days, diagnosis among one’s acquaintances) that may influence risk perceptions and frequency of protective behaviors, and (3) to search for the mediating effects of risk perceptions, anxiety, optimism, fear of death, and social isolation on protective behavior as a function of age. This comprehensive analysis is essential to produce scientific knowledge that may be useful to develop prevention strategies targeting psychosocial dimensions explaining the risk-taking behavior in the early stages of a pandemic, especially in groups at risk for medical complications and mortality due to COVID-19.

## Materials and Methods

### Procedure

A cross-sectional online survey was developed on Qualtrics Software to access the individual responses to the COVID-19 outbreak. The survey was carried out during the first mandatory quarantine for all national citizens during the Emergency State (March 20–April 02). The responses were collected from March 25 to April 02, to allow the collection of the precautionary behaviors from the previous 5 days and after the imposition of quarantine and behavioral restrictions declared by the Government. Considering the recommendations for isolation and to minimize face-to-face interactions, participants were recruited by online advertisements on social media of the university campus and by a snowball sampling strategy, encouraging participants to disseminate the survey to their older relatives and friends. This study was approved by the Local Ethical Committee. All the participants gave informed consent and had the opportunity to read the study information before starting the survey.

### Sample

A total of 1892 subjects participated in this study. However, 174 participants did not complete any COVID-related section of the survey and were removed from the analysis. From the 1718 responses, we additionally excluded 22 participants: 14 included other information than age on the age entry field, two provided atypical response contents, and six completed the survey with an atypical time duration (i.e., less than 2/3 of the expected time).

The final sample included 1696 individuals (30% male) aged between 18 and 85 years old (*M* = 41.9 years, *SD* = 16.5). Most of the sample completed the university (69.9%) or the secondary school (23.9%). For those actively working (62.3%), 69.3% are using teleworking. Only 10.8% of the participants were retired (retirement in Portugal is currently at the age of 66 years and 5 months). The zone of the residence covered all the Portugal mainland geographic regions (North = 61.1%; Central = 22.1%, South = 15.7%) and archipelagos (Madeira and Azores = 0.5% each) and were represented by both rural (23.8%) and city areas (76.2%). Regarding COVID-19, the mean time of quarantine reported by the included participants was of 14.4 days (*SD* = 6.3). 15.5% of the sample reported at least one COVID-related symptom in the past two weeks and 6.0% said they know someone with a confirmed diagnosis. 30.4% disclosed having at least one of the high-risk medical conditions for COVID-19 mortality. 41.1% also reported a past traumatic event. The time of search for and exposure to COVID-related information ranged from less than 1 h to around 1–3 h (89.1%) and was mainly accessed through TV newscasts (88.4%) and social media (80.5%), followed by the reports from the Portuguese Government Health Department (77.1%), newspapers (61.1%), WHO (60.3%), and word of mouth (35.3%).

### Survey Development

The survey collected information on sociodemographic characteristics (e.g., medical conditions, past traumatic experiences) and COVID-19 (e.g., exposure to information, COVID-19 symptoms in the past week, known COVID-19 diagnosis in relatives or close friends). Then, we assessed the perceived risk of COVID-19 based on the estimates of COVID-19 spread compared with the flu (i.e., number of persons who will be contaminated by COVID-19 and the seasonal flu this year in Portugal), COVID-19 contamination (i.e., the probability of becoming infected by COVID-19 in the future and the probability of infecting someone with COVID-19 in the future from a slider ranging from 0 to 100), COVID-19 reactions (i.e., classification of the reaction of the Portuguese Government and of the citizens using response scale ranging from 1 = too extreme to 5 = very insufficient), and penalties for those not following some important practices to mitigate the risks associated with the COVID-19 dissemination (e.g., to go out with COVID-19 active symptoms, do not cover the nose and the mount when someone coughs or sneezes, to host a dinner party at home for friends and familiars, to call to the local urgent health telephonic line to ask how the COVID situation is evolving; the monetary values of the penalties were presented in a slider ranged from 0 to 10.000€).

Regarding behavior, perceived risk was assessed through the classification of high- and low-risk scenarios that were developed based on the local health department and WHO recommendations. Each high-risk scenario was developed to have a corresponding low-risk scenario: (1) to scratch the nose after coming from the street/to scratch the nose after taking bath, (2) to receive visits/to receive supplies at the door, (3) to host a dinner party at home for friends and familiars/to telephone to friends and familiars, (4) to physically compliment someone at the street/to compliment someone at the stress with more than one meter of distance, (5) to go out to meet friends/to go out to practice exercise, (6) to not wash the hands after coming from the street/to not watch hands before waking up, and (7) to use objects that belong to other people/to use personal objects. Participants were asked to move the slider in 0 to 100 scale ranging from “not risky at all” to “very risky.” Additionally, protective behaviors were measured by considering the allowed, but discouraged behaviors during the quarantine as stipulated by a national Decree Law 2-A/2020 of March 20, as well as the most systematically cited protective behaviors by local health authorities [e.g., to buy food and essential supplies (reverse coded), to not physically compliment someone, to wash the hands, to not attend to social events, and to cover the nose and the mount when coughing or sneezing].

Psychological data encompassed self-report measures of state anxiety, optimism, fear of death, and social isolation. Anxiety was measured using the anxiety subscale from the Portuguese version of the Hospital Anxiety and Depression Scale (Pais-Ribeiro et al., 2007; Snaith and Zigmond, 1983). As a measure of state-anxiety we adapted this subscale (six items, α = 0.84) to index the anxiety states specifically related to COVID-19 circumstances (e.g., ‘I feel tense or “wound up” under the actual circumstances;’ “Worrying thoughts about the actual circumstances go through my mind”). Participants were asked to respond in a 4-Lickert Scale where 1 = never and 4 = almost always. Higher scores indicate higher anxiety states related to COVID-19 circumstances. The fear toward the death experience was evaluated by the “fear of death” subscale (seven items, α = 0.90) of the Portuguese Version of the Death Attitude Profile-Revised (Gesser et al., 1988; Serra, 2012). All the items (e.g., “Death is no doubt a grim experience,” “The prospects of my own death arouse anxiety in me”) were rated using a four-Likert scale (1–strongly disagree to 4–strongly agree). Higher scores reveal higher fear of death. The bias toward optimistic outlooks about the future (e.g., “In uncertain times, I usually expect the best”) was assessed through the Portuguese version of the Life Orientation Test-Revised (Laranjeira, 2008; Scheier et al., 1994). This scale includes a total of six items (α = 0.75) rated from a Likert Scale ranging from 1–strongly disagree to 4–strongly agree. Higher scores on this scale index higher optimism about the future. The Portuguese version of the UCLA-Loneliness scale (Russell, 1996; Pocinho et al., 2010) was applied to measure subjective feelings of social isolation in the general life (16 items, α = 0.91, e.g., “I feel isolated from others”), using a Likert scale ranging from 1–never to 4–almost always. Higher scores reflect higher feelings of social isolation in daily life.

The measures included in the survey are described in more detail in Supplementary Table S1.

## Results

Participants were divided into seven age segments, designed to represent the age range in which the daily reports on COVID-19 are nationally presented (18–19, *n* = 126; 20–29, *n* = 420; 30–39, *n* = 233; 40–49, *n* = 280; 50–59, *n* = 350; 60–69, *n* = 208; +70, *n* = 78)^1^. In the next sections we will: (1) analyze group differences in terms of sociodemographic characteristics and COVID-related variables; (2) test for linear and quadratic trends when considering protective behaviors and perceived risk as a function of age; and (3) explore the mediation effects of psychological variables and perceived risk in protective behavior for the different age groups. More details on the analytic strategies are described below.

### Age-Related Groups Differences on Sociodemographic Variables

Supplementary Figure S1 depicts sociodemographic characteristics for each group. Chi-square significant effects were observed for variables which are expected to co-vary with age, namely educational level, *X*^2^(30, 1696) = 335.4, *p* < 0.001, professional status, *X*^2^(18, 1696) = 1980.0, *p* < 0.001, and time of isolation, *F*(6,1654) = 21.8; *p* < 0.001. As expected, there was a higher proportion of participants with high school/university level education and currently active (studying/working) in the younger groups. Moreover, younger adults (18–19) reported to be in isolation for a longer period when compared with all other age groups (all *p* < 0.002). However, there were significant differences between groups for variables that are not expected to be related with age: geographic region, *X*^2^(24, 1696) = 216.6, *p* < 0.001, and sex, *X*^2^(6, 1696) = 26.3, *p* > 0.001. There were more participants in the middle-aged groups from the center and south regions of Portugal. A MANOVA model showed that the effects of geographic regions were significant for protective behaviors, *F*(4,1197) = 669.5, *p* = 0.003, and penalties, *F*(4,1197) = 4.05, *p* = 0.003, with less protective behaviors in the South (i.e., the less affected area; *p* < 0.003), and higher penalties estimates (*p* = 0.001), compared to the North (i.e., the most affected area). Regarding sex, a higher proportion of man was found in the older groups. The effects of sex were more systematic in behavior and risk perceptions (all *p* < 0.039), with men showing less perceived risk and protective behaviors across all variables. For this reason, we included sex as a covariate in the subsequent analyses to correct for its effects. No significant differences were found across age groups on the workplace for those actively employed, *X*^2^(6, 1017) = 8.02, *p* = 0.237, nor rural residence, *X*^2^(6, 1696) = 8.73, *p* = 0.189.

### Age-Related Groups Differences on COVID-Related Variables

Chi-square significant effects were observed for health problems, *X*^2^(6, 1692) = 124.0, *p* < 0.001, trauma, *X*^2^(6, 1696) = 56.3, *p* < 0.001, and symptoms of COVID-19, *X*^2^(6, 1696) = 52.3, *p* < 0.001 (Supplementary Figure S2). There was an increased proportion of participants that experienced at least one symptom of COVID-19 in the younger groups. As expected, there was a higher proportion of participants with health problems and past trauma in the older aged groups. Hypertension and diabetes were the two most prevalent health conditions on the 70+ age group, whereas life-threatening disease and war were the two most prevalent traumatic experiences in this group (Supplementary Figures S3, S4). No significant effects of COVID-19 diagnosis in relatives or close friends were found, *X*^2^(6, 1696) = 7.46, *p* = 0.281. Additionally, results show a significant effect of age on daily time spent on information about COVID-19, *X*^2^(6, 1694) = 73.9, *p* < 0.001, with an increased proportion of participants in the older aged groups spending 1–3 h searching/consuming information about COVID-19 (Supplementary Figure S5). On the younger groups, more than 50% of participants spend less than 1 h searching/consuming information about COVID-19. Of note is that more than 90% of the participants in the older group search for information on TV Newscasts.

### Age Effects on Perceived Risk and Protective Behaviors

Considering the wide age range of our sample and that no assumptions on linear relations between age and both perceived risk and protective behaviors can be definitely withdrawn from literature, we tested whether the results followed a linear, a quadratic, or a cubic trend. The identification of polynomial patterns in data allow to unveil specific linear and curvilinear age-related trajectories in the adoption of protective behavior and perceived risk. Independent univariate ANCOVAS adjusted for sex were conducted with Age (18–19; 20–29; 30–39; 40–49; 50–59, 60–69; +70) as between-groups factor and measures of protective behaviors and perceived risk as the dependent variables. Only the best fit for linear or non-linear trends will be reported. Regression coefficients will be presented to linear effects and Bonferroni comparisons will be described to quadratic effects. All these post-analyses were corrected for sex moderation effects.

### Protective Behaviors

**FIGURE 1:**
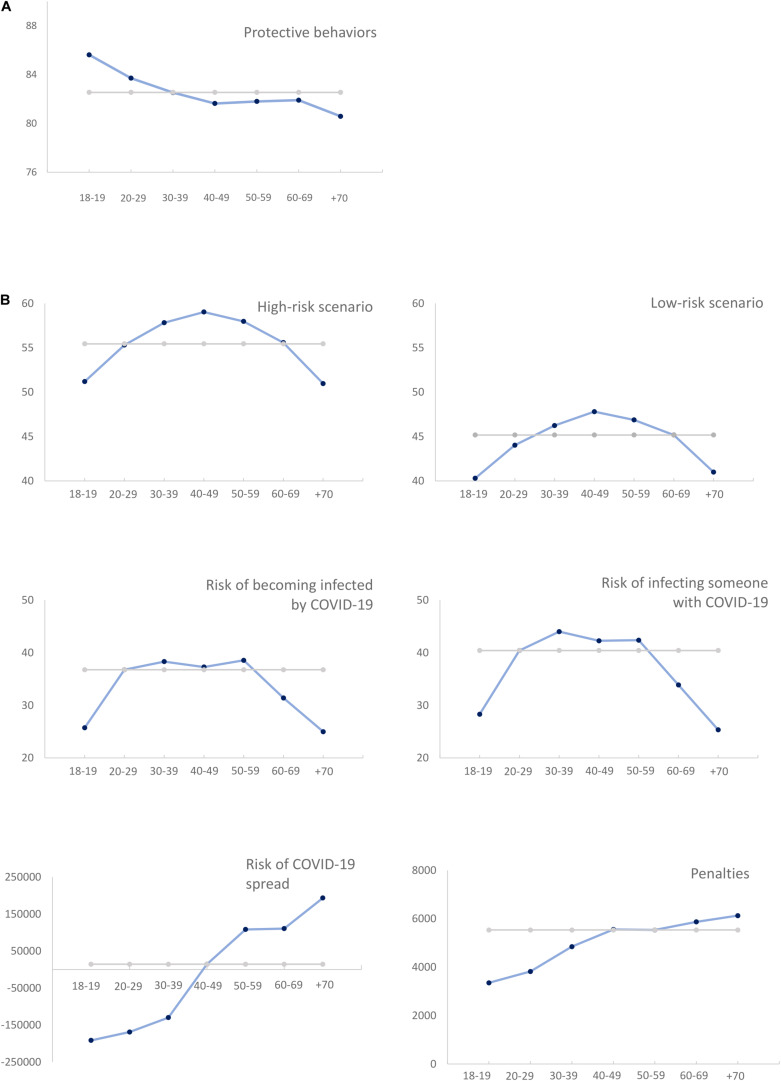
**(A)** Frequency of protective behaviors across age. **(B)** Risk perceptions across age.

The use of protective behaviors (Figure 1A) showed a linear association with age (contrast estimate (CE) = −4.10, S.E. = 1.29, *p* = 0.002), namely for those behaviors allowed but discouraged under the quarantine regulation (CE = −4.04, S.E. = 1.58, *p* = 0.010) and for those encompassing good practices systematically recommended by the local health authorities (CE = −4.16, S.E. = 1.63, *p* = 0.011). Age predicted total scores on protective behaviors (β = −0.097, *p* < 0.001), by indicating a negative association for both quarantine (β = −0.095, *p* < 0.001) and health recommendations (β = −0.062, *p* = 0.017).

### Perceived Risk

As expected, high risk scenarios (*M* = 56.3, *SD* = 16.6) had higher perceived risk, *t*(1567) = 42.8; *p* < 0.001, than the low risk scenarios (*M* = 45.3, *SD* = 13.7). Participants further underestimated, *t*(1646) = −11.2; *p* < 0.001, the probability of becoming infected (*M* = 35.5, *SD* = 22.5) compared to the probability of infecting someone (*M* = 39.3, *SD* = 26.2).

The results revealed an age-related U-inverted quadratic trend for perceived risk in high- (CE = −7.94, S.E. = 1.49, *p* < 0.001) and low-risk scenarios (CE = −7.00, S.E. = 1.23, *p* < 9.001), as well as for the perceived risk of becoming infected by COVID-19 (CE = −13.76, S.E. = 2.00, *p* < 0.001) or contaminating someone with COVID-19 (CE = −17.45, S.E. = 2.32, *p* < 0.001). Lower risk ratings in high-risk scenarios was found for the elders aged above 70, comparing to the 40–49, *p* = 0.006, and the 50–59 age groups, *p* = 0.027 (Figure 1B). No differences were found in relation to other groups (all *p* > 0.052). The same pattern was found for the low-risk scenarios (all *p* > 0.101), expect for the 40–49, *p* = 0.004, and 50–59 groups, *p* = 0.022. The perceived risk of becoming infected or to infect someone had, respectively, less scores on the +70 group compared to adults aged between 20 and 59 years (all *p* < 0.001). Again, the oldest group was not significantly different than the 18–19 and the 60–69 (all *p* > 0.341) (Figure 1B). A quadratic trend with a U-inverted shape further fitted the age effects on perceived (over)reactions (CE = −0.16, S.E. = 0.059, *p* = 0.007). However, when correcting for multiple comparisons, no differences were detected between groups (all *p* > 0.955).

Age effects on the perceived threat of COVID spread in relation to seasonal flu (CE = 368897, S.E. = 128409, *p* = 0.006) and monetary penalties followed a linear trend (CE = 2476, S.E. = 271, *p* < 0.001) (Figure 1B). Age predicted higher perceived threat of COVID-19 spread (β = 0.103, *p* < 0.001), and increased monetary penalties (β = −0.097, *p* < 0.001).

### Mediation Models

A mediation analysis was conducted to assess the mediation effects of psychological processes (mediator 1) and perceived risk (mediator 2) in predicting the total score of protective behavior (dependent variable) across different age groups (independent variable).

On the previous section, the effect of age was linearly associated with protective behaviors while quadratic trends emerged in risk perceptions. To better assess non-linear patterns of results, age was entered in the mediation model as a multicategorical indicator.

Risk perceptions were entered in the model with the status of mediator 2, because risk perceptions can both be modulated by psychological factors (mediator 1) and modulate protective behavior. Anxiety correlated with high perceived risk in high- and low-risk scenarios, and less perceived overreactions (all *p* < 0.019); fear of death showed the same associations with these variables and also with higher penalties for COVID-related transgressions, and higher perceived risk of becoming infected or infecting someone with COVID-19 (all *p* < 0.003); social isolation also covaried with these later perceptions, as well as less perceived risk for COVID-19 spread (all *p* < 0.031). In turn, optimism was related to less perceived risk of becoming infected or infecting someone with COVID-19 (all *p* < 0.001). However, after accounting for the shared variance between the set of risk perception dimensions with significant associations with age, only the perceived risk on high-risk scenarios predicted the higher frequency of protective behaviors (β = 0.112, *p* = 0.016). For this reason, only high-risk perceptions proceeded to the subsequent mediation analysis. This association remained significant in the four mediation models (Tables 1–4).

**TABLE 1:** Mediation Model with anxiety and perceived risk as mediators of the age group–protective behaviors relation.

	**Age → Anxiety**	**Age→ Perceived Risk**	**Age→ Protective Behaviors**	**Age→ Anxiety→ Protective Behaviors**	**Age→ Perceived Risk→Protective Behaviors**	**Age→ Anxiety → Perceived Risk→ Protective Behaviors**
70 vs. 18–19	0.014	0.564	−5.58***	−0.001 [−0.11; 0.13]	0.046 [−0.56; 0.56]	0.004 [−0.07; 0.07]
70 vs. 20–29	–0.041	–3.59	−3.56	0.002 [−0.10; 0.13]	−0.293 [−0.95; 0.16]	−0.013 [−0.07; 0.04]
70 vs. 30–39	–0.123	−6.41**	−2.40	0.006 [−0.16; 0.21]	−0.552 [−1.30; −0.02]*	−0.040 [−0.12; 0.01]
70 vs. 40–49	–0.105	−7.13**	−0.856	0.005 [−0.15; 0.18]	−0.581 [−1.35; −0.63]*	−0.034 [−0.11; 0.02]
70 vs. 50–59	−0.222*	−4.59*	−0.779	0.011 [−0.26; 0.30]	−0.374 [−1.08; 0.10]	−0.071 [−0.17; −0.01]*
70 vs. 60–69	−0.201*	–2.48	−0.546	0.010 [−0.25; 0.29]	−0.202 [−0.86; 0.28]	−0.067 [−0.01; −0.16]*
Anxiety	–	3.95***	−0.050	–	–	–
Perceived Risk		–	0.082***	–	–	–

*p < 0.050, **p < 0.01, ***p < 0.001, [CI 95%].

**TABLE 2:** Mediation Model with optimism and perceived risk as mediators of the age group–protective behaviors relation.

	**Age→ Optimism**	**Age→ Perceived Risk**	**Age→ Protective Behaviors**	**Age→ Optimism→ Protective Behaviors**	**Age→ Perceived Risk→ Protective Behaviors**	**Age→ Optimism→ Perceived Risk→ Protective Behaviors**
70 vs. 18–19	0.045	–0.269	−5.49***	0.085 [−0.32; 0.51]	−0.022 [−0.70; 0.52]	−0.002 [−0.02; 0.01]
70 vs. 20–29	–0.072	−4.95*	−2.75	−0.137 [−0.54; 0.16]	−0.402 [−1.14; 0.07]	0.004 [−0.01; 0.03]
70 vs. 30–39	–0.116	−7.99**	−1.53	−0.220 [−0.70; 0.08]	−0.649 [−1.53; −0.09]*	0.006 [−0.01; 0.03]
70 vs. 40–49	−0.174*	−8.84***	0.019	−0.330 [−0.85; −0.01]*	−0.712 [−1.60; −0.15]*	0.009 [−0.01; 0.04]
70 vs. 50–59	–0.128	−6.53**	0.115	−0.242 [−0.71; 0.04]	−0.531 [−1.34; −0.02]*	0.007 [−0.01; 0.03]
70 vs. 60–69	–0.108	–5.53	0.168	−0.205 [−0.68; 0.10]	−0.368 [−1.12; 0.13]	0.006 [−0.01; 0.03]
Optimism	–	–0.666	1.89**	–	–	–
Perceived Risk		–	0.081***	–	–	–

*p < 0.050, **p < 0.010, ***p < 0.001, [CI 95%].

**TABLE 3:** Mediation Model with fear of death and perceived risk as mediators of the age group–protective behaviors relation.

	**Age→ Fear of Death**	**Age→ Perceived Risk**	**Age→ Protective Behaviors**	**Age→ Fear of Death→ Protective Behaviors**	**Age→ Perceived Risk→ Protective Behaviors**	**Age→ Fear of Death→ Perceived Risk→ Protective Behaviors**
70 vs. 18–19	−0.363**	0.707	−5.35*	0.071 [−0.27; 0.50]	0.060 [−0.56; 0.61]	−0.072 [−0.19; −0.01]*
70 vs. 20–29	−0.257*	–5.25	−2.89	0.050 [−0.21; 0.36]	−0.363 [−1.05; 0.12]	−0.051 [−0.14; 0.01]
70 vs. 30–39	–0.168	−7.70**	−1.85	0.033 [−0.16; 0.30]	−0.658 [−1.47; −0.09]*	−0.034 [−0.12; 0.02]
70 vs. 40–49	–0.057	−8.65***	−0.411	0.011 [−0.13; 0.20]	−0.739 [−1.61; −0.15]*	−0.011 [−0.08; 0.05]
70 vs. 50–59	–0.043	−6.26**	−0.119	0.008 [−0.14; 0.18]	−0.535 [−1.29; −0.02]*	−0.009 [−0.08; 0.05]
70 vs. 60–69	–0.045	–4.85	−0.051	0.009 [−0.14; 0.19]	−0.415 [−1.15; 0.10]	−0.009 [−0.08; 0.05]
Fear of Death	–	2.34***	−0.195	–	–	–
Perceived Risk		–	0.085***	–	–	–

*p < 0.050, **p < 0.010, ***p < 0.001, [CI 95%].

**TABLE 4:** Mediation Model with social isolation and perceived risk as mediators of the age group–protective behaviors relation.

	**Age→ Social Isolation**	**Age→ Perceived Risk**	**Age→ Protective Behaviors**	**Age→ Social Isolation→ Protective Behaviors**	**Age→ Perceived Risk→ Protective Behaviors**	**Age→ Social Isolation→ Perceived Risk→ Protective Behaviors**
70 vs. 18–19	–0.165	–0.278	−5.85**	0.374 [0.01; 0.85]*	−0.022 [−0.63; 0.46]	−0.002 [−0.03; 0.03]
70 vs. 20–29	–0.148	−4.63*	−3.77*	0.335 [0.03; 0.74]*	−0.371 [−1.05; 0.08]	−0.002 [−0.02; 0.02]
70 vs. 30–39	–0.110	−7.69**	−2.55	0.250 [−0.07; 0.66]	−0.616 [−1.43; −0.09]*	−0.001 [−0.02; 0.02]
70 vs. 40–49	–0.107	−8.22***	−1.11	0.243 [−0.05; 0.64]	−0.659 [−1.50; −0.12]*	−0.001 [−0.02; 0.02]
70 vs. 50–59	–0.100	−6.27*	−0.892	0.226 [−0.06; 0.58]	−0.502 [−1.24; −0.02]*	−0.001 [−0.02; 0.02]
70 vs. 60–69	−0.211*	–4.06	−0.900	0.477 [0.11; 0.95]*	−0.325 [−1.02; 0.14]	−0.002 [−0.03; 0.03]
Social Isolation	–	–0.013	−2.26***	–	–	–
Perceived Risk		–	0.080***	–	–	–

*p < 0.050, **p < 0.01, ***p < 0.001, [CI 95%].

From this analytic strategy, four independent models for anxiety, optimism, fear of death, and social isolation were carried out on PROCESS v3.4 (Hayes, 2012) using the mediation model nr. 6 with X = Age groups (defined as a multicategorical variable with +70 age group as the reference group), M1 = psychological dimensions, M2 = perceived risk, and Y = protective behaviors. Considering that a multicategorical predictor variable contemplates more than one indirect effect (g–1 = 6), the predictor effect on the outcome variable is mediated by a given variable if at least one of the relative indirect effects is different from zero in the respective bootstrap confidence interval for inference (Hayes, 2018).

### Anxiety

The inclusion of anxiety (mediator 1) and perceived risk (mediator 2) as mediators of the age group–protective behaviors relation revealed the following (Table 1): (a) the regression of the age group comparisons with anxiety was significant for the 50–59 (β = −0.352, *p* = 0.013) and the 60–69 age groups (β = −0.330, *p* = 0.028), with less reported anxiety in the +70 compared to these age groups; (b) anxiety positively predicted perceived risk (β = 0.154, *p* < 0.001), and (c) the regression effect of anxiety with the frequency of protective behaviors was non-significant (β = −0.002, *p* = 0.929).

On the mediation effects, the bootstrap CIs for inference about the relative indirect effects of age groups in protective behaviors revealed that anxiety was a non-significant mediator. When considering both mediators in the same model, anxiety and perceived risk mediated the age-related differences in the 50–59 and 60–69 age groups. The indirect effects of anxiety and perceived risk in these groups potentiates the reduced frequency of protective behaviors in the +70-age group, when compared with the 50–59 and 60–69 age groups.

### Optimism

The model exploring the role of optimism (mediator 1) and perceived risk (mediator 2) on the age group–protective behaviors relation showed that (Table 2): (a) age-related differences in the 40–49 age group significantly predicted optimism (β = −0.313, *p* = 0.038), with participants above 70 years reporting less optimism; (b) optimism did not predict risk perceptions (β = −0.023, *p* = 0.385), and (c) optimism was associated with increased protective behaviors (β = 0.079, *p* = 0.003),

On the mediation effects, the bootstrap CIs for inference about the relative indirect effects of age groups in protective behaviors unveiled optimism as a significant mediator in the 40–49 age range, i.e., diminished optimism potentiated the reduced frequency of protective behaviors in the +70-age group when compared with the 40–49 age group. No mediation effects were found for the bootstrap CIs for inference about the relative indirect effects of age groups in protective behaviors when accounting for both mediators.

### Fear of Death

The inclusion of fear of death (mediator 1) and perceived risk (mediator 2) in the model unveiled that (Table 3): (a) the +70 group reported less fear of death than younger individuals (18–19, β = −0.431, *p* = 0.009; 20–29, *p* = 0.035) (b) fear of death significantly predicted high perceived risk (β = 0.122, *p* < 0.001), and (c) the regression effect of fear of death on protective behavior was non-significant (β = −0.012, *p* = 0.648).

Regarding mediation effects, the bootstrap CIs for inference about the relative indirect effects of age groups in protective behaviors indicated that fear of death did not mediate this association. For both mediators, this analysis showed a significant indirect effect in the 18–19 age group. Data suggest that reduced fear of death along with reduced risk perceptions in the +70-age group reduce the engagement in protective behaviors in the +70-age group when compared with the 18–19 age group.

### Social Isolation

Accounting for the mediation effect of social isolation (mediator 1) and perceived risk (mediator 2) on the age group–protective behaviors relation, it was found that (Table 4): (a) the regression of the age group comparison with social isolation was significant for the 60–69 group (β = −0.379, *p* = 0.014), with the +70 reporting less social isolation; (b) the regression effect of social isolation on perceived risk was non-significant (β = −0.001, *p* = 0.987), (c) but social isolation predicted reduced protective behaviors (β = −0.095, *p* < 0.001).

The analysis of the bootstrap CIs for inference about the relative indirect effects of age groups in protective behaviors showed that social isolation mediated age effects on protective behavior for the 18–19, 20–29, 60–69 age clusters. The indirect effects suppressed the main effect of age on protective behaviors, suggesting that social isolation reduces the frequency of protective behaviors in younger (i.e., 18–19; 20–29) and older adults (i.e., 60–69) when compared with the +70 age group. The model including the two mediators did not revealed any significant mediation effect across age groups.

## Discussion

The first responses to a pandemic are inevitable preventive and are highly dependent on the individual reactions. Therefore, as the outbreaks spread across the globe, there is an urgent need for psychological studies gathering evidence on variables that may influence protective behaviors, namely for those groups who are at high-risk. From a scientific standpoint, it is unknown how older adults are reacting to an unexpected situation that requires sudden modifications of routines. This study represents an effort to analyze risk perceptions and the frequency of protective behaviors in older adults during the first days of the outbreak while exploring group differences that may underlie these variables.

### Protective Behaviors

Overall, the results show that protective behaviors decline with advancing age. Specifically, older adults seem to engage more in those routine behaviors that are strongly discouraged during the quarantine, regardless of being allowed, and to engage less in those health practices recommended to prevent the contamination. Considering that younger groups reported longer isolation periods, we should equate whether group differences in protective behaviors are related to specific differences in seeking essential goods from services that remained open during the quarantine (e.g., markets, pharmacy, etc.). However, it is not possible to simply attribute the older adults’ risk-taking behavior to the active management of the household, since older adults also engaged less in prevention measures related to health practices aiming to prevent infection (e.g., to wash the hands, or to cover the nose and the mouth when coughing or sneezing). Accordingly, the older group was less likely to follow the protective recommendations in previous SARS (Wong and Tang, 2005) and H1N1 pandemics (Rubin et al., 2009), where they were also at higher risk. This is of high importance, given that the current older adults’ sample had more health problems that relate to risk for medical complications and mortality, namely hypertension and diabetes.

The interplay between group differences in sociodemographic characteristics, COVID-related variables, risk perceptions, and psychological dimensions will be explored below to provide the comprehensive insight on risk and protective factors that may affect the adoption of preventive measures.

### Sociodemographic Characteristics and COVID-Related Variables

The evidence shows that older adults exhibit some protective factors for risk assessment and preventive attitudes. Considering their greater health vulnerability and higher exposure to information from TV newscasts, it would be expected an increased frequency of protective behaviors (Leung et al., 2003; Wang et al., 2020). No group differences were found in COVID-19 diagnosis among acquaintances. Nonetheless, older adults reported other aspects that represent potential risk factors: this group was most likely to be retired, to have lower educational levels, and to report higher traumatic experiences (Fielding et al., 2005; Bish and Michie, 2010).

Although TV newscasts dedicate a significant part of the airtime to daily reports from the local health authorities, there are expressions systematically repeated that may not be very intelligible to individuals with lower educational levels (e.g., “exponential curve” or “asymptomatic case”). Thus, communication strategies toward health education not only need to be designed to reach and target vulnerable groups (i.e., older and/or risk-taking adults) as also need to use accessible messages for those with lower educational levels. Retirement can also contribute to blurring the significance and urgency of the problem, since a detachment of current issues, or at least a delay in risk perception, is expected when people furthest from the everyday workplace discussions around preventive measures and the possibility of wind up activities. Finally, the traumatic experiences in older adults, namely life-threatening diseases, along with reduced reported symptomatology related with COVID-19, can desensitize for the relevance of the problem. Fielding et al. (2005) previously stated that hazard familiarity in older adults may interact with risk assessment during outbreaks.

### Risk Perceptions

Participants were capable of distinguishing high-risk from low-risk scenarios. In both scenarios, an inverted U-shape revealed that older adults (i.e., 60–69 and +70) are not significantly different from younger adults (i.e., 18–39) in risk assessment of scenarios encompassing high (i.e., to scratch the nose after coming from the street) and low-risks (i.e., to scratch the nose after taking bath) and that these two groups perceive less risk than middle-aged adults (i.e., 40–59). Interestingly, participants underestimated the probability of becoming infected compared to the probability of infecting someone, which is paradoxical but strengthens the assumption that individuals tend to see their chances of having health problems as lower than their peers (Weinstein, 1984; Chapin, 2001; Sharot, 2011). These probabilities followed the same U-inverted shape, with the youngest and the oldest groups (i.e., 18–19 and +70) underestimated the probabilities of becoming infected and of infecting someone. From these findings, both older and younger adults estimated less the individual risk, but only older adults showed reduced protective behaviors. These results are in accordance with studies showing that elders perceive lower risks in epidemics (Fielding et al., 2005; Bish and Michie, 2010) and suggest that middle-aged adults are more accurate in risk assessment.

Nevertheless, an opposite pattern was found in risk estimates related to COVID-19 spread. Age linearly predicted increased threat estimates for COVID-19 spread and higher penalties for those not following practices preventing the COVID-19 dissemination. Taken together, our results reveal that older adults appear to be aware of the general COVID-19 threat, but these risks seem to be underestimated when they are assessed at individual and more specific behavioral levels. Of note, lower ratings on high-risk scenarios uniquely predicted reduced engagement in protective behaviors, and older adults showed reduced perceived risks in these scenarios. As such, the response from this group to the outbreak seems to rely specifically on individual risk perceptions. This suggests that subjective beliefs about preventive measures should not be disregarded and that public health messages should be very clear about the outbreak risks in order to reduce subjective interpretation, namely by providing adequate and objective messages targeting those who are at high-risk. This is of high relevance because older adults seem to be capable lo learn under uncertain contexts of decision-making and move to decisions based on known outcomes (i.e., decision based on risk), albeit in a less effective way than younger adults (Pasion et al., 2017; Fernandes et al., 2018).

### Psychosocial Dimensions

The oldest group (+70) reported lower state-anxiety levels associated with the COVID-related circumstances and lower fear of death than 50–69 and 18–29 age groups, respectively. These results, collected during an unfamiliar situation with an ongoing rampant health crisis, are in the same vein of the positivity effect in aging (Charles and Carstensen, 2010; Mather, 2016): negative affect–such as anxiety symptoms (Kessler et al., 2005; Flint et al., 2010) and fear of death - declines with aging (Fortner et al., 2000; Cicirelli, 2002; Russac et al., 2007; Thorson and Powell, 2000). Anxiety predicted higher perceived risk and showed to be a protective factor for adopting preventive behaviors in the 50–59 and 60–69 age groups, when compared to participants aged above 70. A similar pattern was found regarding fear of death: this dimension was associated with higher perceived risk and worked as a protective factor for engaging in preventive behaviors in the younger sample (i.e., 18–19). In turn, lower anxiety and fear of death coupled with impaired risk perceptions might restrain the frequency of protective behaviors in the +70-age group. These effects were specifically mediated by ratings in high-risk scenarios, but it should be considered that fear of death further correlated positively with larger penalties amounts for transgressions and heightened perceived risk of becoming infected or infecting someone. Both anxiety and fear of death were also associated with lower ratings on overreactions from Government and citizens.

From these results, moderated levels of anxiety and fear of death may increase protective behaviors via higher perceptions of risk. That is, moderated levels of anxiety and fear of death may be adaptive by potentiating a defensive response in situations where survival is at risk. The effect of anxiety on protective behaviors was previously observed in SARS, H1N1, and H5N1 outbreaks (Leung et al., 2003; Vijaya et al., 2005; Rubin et al., 2009; Bish and Michie, 2010), particularly due to higher risk perceptions in anxious individuals (Fieldman et al., 2005; Vijaya et al., 2005). However, it should be acknowledged that excessive anxiety and fear of death can trigger panic reactions that are highly disruptive for the mental well-being. For instance, clinical chronic anxiety is essentially different from reactive anxiety patterns toward cautionary measures when it does not interfere significantly with daily life and requires different intervention strategies to cope with. Considering the complexity of the phenomenon, psychologists might also take a pivotal role in multidisciplinary teams when developing strategies to manage risk perceptions in a way that does not disregard the mental well-being and, simultaneously, promotes cautionary behaviors. These strategies must also equate for the habituation effects of exposure to repetitive messages.

Optimism was included in the analysis as a positive affective outlook about the future that may compromise the engagement in protective behaviors by reducing the perceived risks, especially in older adults due to the positivity bias. Previous studies found that persons are likely to underestimate the risks of becoming infected by diseases such as SARS (Brug et al., 2004) and COVID-19 (Wise et al., 2020), even when compared to non-infectious medical conditions (e.g., cancer and heart attack). Despite associations of optimism with underestimates of becoming infected or infecting someone with COVID-19, the current study found that higher optimism predicted directly the adoption of preventive measures in the 40–49 group when compared to the oldest group who were less optimistic about the future. As such, the current study did not found evidence for optimism as a risk factor for older adults’ risk-taking behavior. On the contrary, reduced optimism in the +70-age group may potentiate a decline in preventive measures when compared with the 40–49 age group.

The protective role of optimism brings interesting possibilities to counterbalance negative and positive affect when managing both risk perceptions in the adoption of preventive measures and the broad individual reactions to COVID-19 circumstances (e.g., self-isolation). The inclusion of optimistic perspectives about the future, namely during psychological interventions, may help to manage expectations toward a reality that is inherently aversive in the short-term, but necessary to avoid the spread of the virus and to return to the (new) normality in the medium-term. Our brain may not be accurate when making inferences about the future (Weinstein, 1984; Sharot, 2011), especially in what regards health problems (Chapin, 2001)–and that is why communication on health issues needs to be clear about the risks -, but an optimistic mindset may be adaptive to overcome adversities. In fact, optimistic messages rapidly echoed worldwide: *andrá tutto bene*, everything will be alright.

Finally, we explored the subjective experiences of social isolation. Social isolation covaried with estimates of becoming infected or infecting someone and with lower perceived risk for COVID-19 spread. Nevertheless, social isolation predicted protective behaviors such as optimism did (i.e., only anxiety and fear of death seem to modulate prevention attitudes via perceived risk). Specifically, social isolation decreased the frequency of protective behaviors in the 18–19, 20–29, and 60–69 age groups, inversely to what was found for optimism. Despite the reduced levels of social isolation reported by the +70 participants, these findings show that social isolation is a risk factor for risk-taking behavior, namely in older and younger adults.

There is evidence that individuals lacking social support are less exposed to multiples sources of information and normative pressures from their peers, and may be less motivated to adhere to socially defined standards (Cacioppo and Hawkley, 2003; Lauder et al., 2006; Hawkley and Cacioppo, 2010). Additionally, these individuals might be more likely to interrupt the quarantine to get essential goods and supplies and, consequently, might be more exposed to COVID-19 (Jones, 2020). This may be more critical for adults aged above 60 years, but younger adults are also active routes of transmission, and therefore, highlight the need for appropriate social responses. Of importance, the relationship between social isolation and health-promoting behaviors seem to not rely exclusively on objective indexes (e.g., quality of the social network). In accordance with previous studies, our results demonstrate that this link is also dependent on the subjective feelings of social isolation and loneliness (Lauder et al., 2006; Hawkley and Cacioppo, 2010; Holt-Lunstad et al., 2015; Hämmig, 2019). Psychologists are in a privileged position to flag those individuals lacking social networks or reporting higher feelings of loneliness. For example, community psychologists that contact with social excluded groups. Thus, these professionals may assess the social support network of these individuals and in cases where this network is manifestly insufficient activate strategies to cope with this specific situation and minimizing risk-taking behaviors.

### Limitations

Some limitations should be acknowledged. First, the present study provides a cross-sectional analysis, and data were collected in a single moment during the early stages of the COVID-19 outbreak in Portugal. As so, no follow-up analysis was conducted on how the evolution of the outbreak changes individuals’ perceptions and behavior, no causal inferences can be drawn on the mediation effects, and results may not be generalized to other countries, namely those with different approaches to target the COVID-19 pandemic.

This procedure further limited recruitment opportunities and sample size but allowed for circumscribing risk-taking behaviors and perceptions to the first phases of the outbreak. Of note, unbalanced groups did not statistically lead to group size distributions affecting variance of the error distribution, which suggest that group size differences did not affect the overall findings. Second, some carryover effects may be present, especially in optimism and social isolation scales that were administered after the state anxiety measures. Third, the procedure (i.e., survey) and online data collection may have biased the included sample and limits the generalization of findings. For instance, surveys show several drawbacks (e.g., social desirability, subjective interpretations) and individuals with access to technologies may be fundamentally different from those who do not have frequent access to computers, smartphones, and internet, namely in what regards age (i.e., people over 70 years old that have experience with technology may be different from those older adults with no access to technology). However, the quarantine circumstances limited the available options for data collection and, even so, this study was able to find the sociodemographic characteristics that are expected to co-vary with age (e.g., education). Finally, variables related to information exposure and psychosocial dimensions gave important insights. Nonetheless, a more fine-grained analysis on information variables (e.g., effective knowledge and information acquired through public health communications) and the inclusion of other psychological dimensions (e.g., hypochondria symptoms and compulsive cleaning behaviors) would allow for a more comprehensive picture. Also, it is not possible to accurately measure from survey procedures that the reported social media exposure corresponds linearly to the effective attention allocated to the COVID-related news.

## Conclusion

The present study provides a comprehensive analysis on the sociodemographic and psychosocial accounts for the age effects on risk perceptions and protective behaviors during the early stages of the COVID-19 outbreak. Since an effective response during the early stages of an outbreak is paramount for a successful containment and control of the contagion, the present study provides valuable information for public health policies, in order to promote protective behaviors among particularly vulnerable groups. Results show that the engagement in protective behaviors declines with advancing age and that older adults show a pattern toward lower perceived risk compared with middle-aged adults. They further evidence that anxiety, optimism, fear of death, and social isolation significantly mediate age effects on protective behaviors. Specifically, both anxiety and fear of death increase protective behaviors via higher perceived risk in the middle-aged and in the younger groups, respectively. Optimism directly predicts protective behaviors in the middle-aged groups, while social isolation reduces protective behaviors in the younger and older-aged groups. Therefore, attention should be given not only to the study of the effectiveness of public health communications directed to groups at risk, but also to mental health, as psychosocial variables such as anxiety, optimism, fear of death and social isolation account for age differences in the adoption of protective behaviors. Mental health practitioners, especially psychologists, are also challenged by the current crisis, providing interventions mainly based on digital solutions. The results of the present study provide elucidation on potential risk factors for disruptive behaviors during pandemics, that are fundamental for an effective communication and intervention that promote both protective behaviors and mental health.

## Data Availability Statement

The raw data supporting the conclusions of this article will be made available by the authors, without undue reservation.

## Ethics Statement

The studies involving human participants were reviewed and approved by the Local Ethical Committee of the Faculty of Psychology and Educational Sciences, University of Porto. The patients/participants provided their written informed consent to participate in this study.

## Author Contributions

All authors contributed to conceptualization and manuscript review. RP, CF, and TP: data collection. RP, TP, and FB: data analysis. RP and CF: manuscript preparation. FB: supervision.

## Conflict of Interest

The authors declare that the research was conducted in the absence of any commercial or financial relationships that could be construed as a potential conflict of interest.

## Footnotes

1 Levene’s Homogeneity tests results reveal homogeneity in variances across groups for Anxiety and Optimism (*p* > 0.05). For the frequency of protective behaviors, risk perception, isolation, and fear of death, results show that in spite Levene’s test revealing unequal variances (*p* < 0.05), the White’s test for Heteroskedasticity for all variables suggest that the variance of the errors does not depend on the group size distribution (all *p* > 0.05).
